# Intermittent Fever, Part 1b

**Published:** 1891

**Authors:** P.P. Wells


cold sweat on the forehead; first heat and sweat, then chill with shuddering and chattering of the teeth.

4. Sweat with the heat; sweat after the chill without heat.

**Carbo-animalis.**

1. Pulse excited and accelerated, with throbbing in the vessels, most toward evening.

2. Chill, especially afternoons, after eating, and evenings; shuddering chill every other day in the evening and continuing after he was in bed; evening chill, with subsequent sweat in sleep.

3. Heat after each preceding chill, mostly at night in bed.

4. Sweat after the heat, usually toward morning; sweat in the day from the slightest movement, even from eating; nocturnal sweat, which is offensive, debilitating, and stains the linen yellow; sweat most abundant on the thighs.

**Carbo-vegetabilis.**

1. Pulse weak and languid, often imperceptible; intermittent pulse; irregular pulse, then much accelerated, then again as if suppressed.

2. Chill and coldness, mostly evenings, usually with thirst, sometimes only one-sided—the left; during the chill, uncommon weakness; chill, with icy coldness of the body.

3. Heat after the chill, evenings or nights in bed, with many concomitant symptoms; evening attacks of flying, burning heat, usually without thirst.

4. Copious sweat, mostly offensive or sour smelling; great disposition to sweating, even while eating; night-sweats; sour smelling sweat in the morning.

**Causticum.**

1. Pulse somewhat excited toward evening from blood ebullition.

2. Chill and coldness predominant, often with coldness of the whole left side; great internal chill, with accompanying sweat, without precedent heat; strong internal chill about midnight; shuddering proceeding from the face.

3. Heat in the evening from six to eight o'clock; running heat over the body, and then chill.

4. Sweat immediately after the chill, without preceding heat; great sweat while going in the open air; sour smelling night-sweat; morning sweat about four o'clock.

**Chamomilla.**

1. Pulse small, but tense and accelerated; often very irregular, and then for a time weak.

2. Chill and shuddering, usually only on single parts, with heat in blood-vessels; shuddering chill, with internal heat; chill and coldness of the whole body, with burning hot face and hot breath; alternating shuddering and chill of some parts, with heat of others; chill of the back part, with heat of the front of the body, or the reverse; cold shuddering with each uncovering and in the cold air.

3. Heat mingled with cold shuddering, mostly with one red and one pale cheek; anxious heat with sweat on the face and hairy scalp; long-continued heat, with great thirst and frequent waking from sleep in a fright.

4. Sweat in sleep, greatest on the scalp, mostly sour smelling and with smarting of the skin; repelled sweat, and then it is wholly wanting.

**Chelidonium.**

1. Pulse small and rapid; fuller, harder, but little accelerated pulse toward evening.

2. Chill and chilliness, only internal, with strong shaking, in the evening, in bed; internal chill while going in the open air, which disappears in a room; chill and coldness of the whole body, most on the hands and feet, with great swelling of the veins; chill of one (right) leg below the knee; shuddering, without external coldness; shuddering on the back, running downward.

3. Internal heat, without thirst, evenings after lying down.

4. Sweat during sleep, after midnight, and in the morning on waking, which soon disappears.

**China.**

1. Pulse small, but hard and quick, after eating more quiet; pulse irregular and sometimes intermittent; uncommon swelling of the veins.

2. Chill over the whole body, increased by drinking, with thirst before or after, and not during the chill; internal severe chill with ice-cold hands and feet, with rush of blood to the head; alternating chill and heat in the afternoon; in the evening he cannot get warm in bed.

3. Heat over the whole body, with swollen veins; during the heat (as with the chill), thirstlessness, or merely thirst for cold drinks; after the heat, severe thirst; long-continued heat, which often appears long after the chill; during the heat, disposition to uncover himself.

4. Great and debilitating sweat; easy sweating in sleep and moving in the open air; very debilitating night or morning sweat; sweat is often fatty or cold; increased thirst with the sweat; repelled and wanting sweat; sweat on the side on which one lies.

**Cicuta-virosa.**

1. Pulse weak, slow, and trembling, sometimes wholly wanting.

2. Chill and chilliness, with longing for warmth and the warm stove; the chill goes from the chest and runs downward to the legs and to the arms, with rigidity.

3. Heat slight, and only internal.

4. Sweat night and morning, most on the abdomen.

**Cina.**

1. Pulse small, but hard and accelerated.

2. Chill, with shuddering and shaking, running from the upper part of the body to the head, even by a warm stove; chill, with coldness of the pale face, and warm hands; chill not to be relieved by external warmth, mostly in the evening, with great paleness of the face.

3. Heat greatest on the head and face, but with great paleness of the face; nocturnal heat with thirst.

4. Sweat, generally cold, on the forehead, nose, and hands; after the sweat, which often precedes the beginning of the chill, vomiting of food, and at the same time a ravenous appetite.

**Clematis-erecta.**

1. Pulse excited, with throbbing in all the blood-vessels.

2. Chill with shuddering, then sweat, without preceding heat; shuddering chill from every uncovering.

3. Dry heat, with general sensation of heat, only at night.

4. Great sweating, mostly at night and morning, with aversion to uncovering.

**Cocculus.**

1. Pulse small, jerking, often imperceptible, seldom hard, and somewhat accelerated.

2. Chill frequently alternates with heat; internal chill, with shuddering, afternoon and evening, over the whole body, most on back and legs, not to be relieved by external heat; constant chilliness, with hot skin.

3. Dry heat through the whole night; flying heat, with burning heat of the cheeks and cold feet.

4. Sweat the whole night, which is cold only on the face; morning sweat, most on the chest; debilitating sweat over the whole body from the slightest movement; sweat on the painful parts.

**Coffea-cruda.**

1. Pulse generally wholly unaffected, and only very little accelerated.

2. Chill increased from each beginning of motion; frequently recurring internal shuddering chill, with external heat of the face or of the whole body; chilly sensation, with internal or external warmth; great sensitiveness to cold air; chill runs down the back.

3. External, dry heat, evening after lying down, with shuddering on the back; nocturnal, dry heat, with delirium; great heat of the face; hot breath.

4. Sweat sometimes after the heat; slight morning sweat; sweat on the face, with internal, cold shuddering.

**Colchicum.**

1. Pulse uncommonly accelerated, hard and full.

2. Chill and shuddering running through all the limbs; frequent shuddering chills running down the back.

3. Only external, dry heat of the skin; dry heat through the whole night, only external, with great and unextinguishable thirst.

4. Sweat wholly suppressed and wanting.

**Colocynth.**

1. Pulse generally full, hard, and accelerated, seldom small and weak; strong throbbing in all the blood-vessels.

2. Chill and coldness of the whole body, often with heat of the face; either cold hands or soles of the feet, with general warmth of the body; chill and shuddering with the pains.

3. External dry heat; internal sensation of heat, with attacks of flying, external heat.

4. Night-sweat, of urinous smell, which causes itching of the skin; sweat, especially on the head and extremities.

**Conium-mac.**

1. Pulse extremely irregular, mostly slow and large, with intermingled small and quick beats; sensible pulsations in the blood-vessels of the whole body; entire pulselessness.

2. Chill and coldness mornings and afternoons (from three to five o'clock); chill, with constant desire for warmth, especially that of the sun; mornings only internal coldness; afternoons with running shuddering.

3. Great heat, internal and external, with great nervous excitability; heat, with concomitant, copious sweat.

4. Sweat day and night, as soon as one sleeps, or even closes his eyes; night and morning sweat, which is offensive, and causes smarting of the skin.

**Creosotum.**

1. Pulse small and weak, with great ebullition of blood; in repose all the blood-vessels throb.

2. Chill predominates mostly in repose; shaking chill, with great flashing heat of face, red cheeks, and ice-cold feet; chill, with great bodily restlessness; chill alternating with heat.

3. Heat, mostly in the face; flying heat, with sharply circumscribed redness of the cheeks.

4. Sweat slight and only in the morning, with heat and redness of cheeks.

**Crocus-sativus.**

1. Pulse feverish and accelerated; anxious palpitation of the heart.

2. Chill in the afternoon, increased toward evening, with shuddering chill from the back downward and trembling ; thirst with both chill and heat ; shuddering chill only on the back half of the body.

3. Flying, internal heat, with pricking and crawling in the skin ; heat, most of the head and face, with pale cheeks and thirst ; heat, with great redness of the face and swollen veins.

4. Sweat slight, only in the night, and then cold and debilitating ; sweat only on the lower half of the body.

**Cuprum.**

1. Pulse generally small, almost imperceptible, weak, and very slow ; seldom full, hard, and accelerated.

2. Chill over the whole body, greatest on the extremities ; chill after every attack of illness (also after epilepsy) ; ice coldness of the whole body.

3. Over-running flashes of heat ; debilitating, hectic, internal heat.

4. Cold sweat at night ; many attacks (of epilepsy and mania) end in (cold) sweat.

**Cyclamen-europeum.**

1. Pulse not perceptibly changed.

2. Chill forenoon or evening ; shuddering chill over the whole body, morning or evening ; during the evening chill great sensitiveness to cold air and to being uncovered.

3. After the chill, heat, most of the face, but without thirst, with long-continued cold hands ; sensation of heat in the whole body, especially in face and hands ; heat of single parts, but not of the face ; universal heat after eating.

4. Sweat at night, in sleep, moderate, but offensive.

**Digitalis.**

1. Pulse extremely slow, especially in repose ; pulse irregular and sometimes intermittent ; pulse accelerated greatly, and immediately, by every motion, and is full and hard, but sinks, in repose, soon to its usual slowness.

2. Chill more internal, with warmth of the face, but beginning with coldness of the extremities, from which it spreads over the whole body ; chilliness and shuddering over the whole back; internal chilliness, with external warmth; general chill, with heat and redness of the face; chill and heat alternating; general coldness of the hands and feet, with cold sweat; great sensitiveness to cold.

3. Heat, mostly appearing late after the chill; sudden flying sensation of heat, with subsequent weakness; increased bodily warmth, with cold sweat on the face; heat of one hand, with coldness of the other.

4. Sweat in the night, mostly cold and somewhat sticky; sweat immediately after the chill, without precedent heat.

**Drosera-rotundifolia.**

1. Pulse unchanged.

2. Chill, with coldness and paleness of the face and cold extremities; chill, forenoons; internal chill at night in bed and in repose; in the morning, left side of the face cold, the right hot; chill and shuddering in repose, and all appears too cold to him, even in bed; chill in the day, at night, heat.

3. Heat almost entirely of the face and head; increased heat of the upper part of the body in the evening.

4. Nights, warmer sweat, especially after midnight and in the morning, most on the face.

**Dulcamara.**

1. Pulse small, hard, tense, especially at night.

2. Chill, spreading itself from the back, mostly toward evening, not relieved by external warmth; chill, with the pains; chill, with great thirst.

3. Universal, dry, burning heat over the whole body; heat and burning in the back, heat and delirium, without thirst.

4. Offensive sweat over the whole body, nights and mornings, during the day it is more on the back, epigastrium, and palms of the hands; sweat entirely suppressed.

**Euphorbia-officinarum.**

1. Pulse.

2. Chill and coldness predominate; chill when beginning to eat and while walking in the open air, though the air is not cold; chill, with concomitant sweat; shuddering chill over the whole upper part of the body, with heat of the cheeks; want of proper bodily warmth, with internal, burning heat.

3. Heat with intolerance of bed covering, which seems too heavy; heat only of the head.

4. Sweat in the morning, in bed; cold sweat on the legs.

**Euphrasia.**

1. Pulse unchanged.

2. In the forenoon chill and internal coldness, which in the afternoon is changed to external chill and coldness, especially on the arms; predominant chilliness.

3. Attacks of heat in the daytime, with redness of the cheeks, and cold hands.

4. Sweat at night in sleep, which is very copious and offensive, most abundant on the throat.

**Ferrum.**

1. Pulse full and hard; great ebullition of blood.

2. Shuddering chills in frequent short attacks; fever chill with red, hot face and thirst; general coldness in the evening, in bed, often lasting the whole night; chilliness and want of natural bodily warmth.

3. Dry heat over the whole body, especially toward evening, with great redness of the face and inclination to be uncovered.

4. Copious and long-continued sweat, from every movement in daytime, as well as nights and mornings in bed; sticky, and for the most part debilitating sweat; sweat every other day from morning till noon; strong smelling night-sweat; sometimes cold, anxious sweat (with spasms).

**Fluoric acid.**

1. Pulse only slightly accelerated by motion.

2. Chill entirely wanting.

3. Universal heat with nausea, from the slightest movement, with inclination to be uncovered, but more for cold washing.

4. Sticky, sour and unpleasant smelling sweat, most on the upper part of the body, especially from motion, afternoons and evenings. The sweat favors the appearance of excoriations, especially of parts on which one lies.

**Graphites.**

1. Pulse full and hard, but not noticeably accelerated.

2. Chill and coldness, mostly evenings; chilliness day and night, especially evenings after four o'clock.

3. Universal dry heat through the evening and night, after a preceding chill; heat while riding in a carriage.

4. Sweat from the least movement; copious night-sweat; sweat sour, offensive smelling, staining the linen yellow, and often cold; utter inability to perspire.

**Helleborus-niger.**

1. Pulse generally small, slow, and hardly perceptible.

2. Chill predominates in the daytime, as often as repeated, with heat of the face; chill alternates with pains in the joints; shaking chill, with goose-flesh and pains in the joints; the shuddering proceeds from the arms.

3. Heat in the evening and daytime, as soon as he lies down, generally accompanied by sweat; burning heat over the whole body, evening, in bed, with shuddering and aversion to drink; first heat, then chill, with pains in the abdomen, in repeated attacks.

4. Sweat in bed, with the heat increased toward morning; cold, sometimes sticky sweat.

**Hepar-sulphur.**

1. Pulse hard, full, and accelerated, sometimes intermittent, with ebullition of blood and throbbing of the veins.

2. Chill, regularly each evening about six or seven o'clock; chill in the daytime in alternation with heat and photophobia; chill at night in bed with aggravation of all the sufferings; great chilliness in the open air.

3. Dry, burning heat, with redness of the face and great thirst the whole night; flying heat, with sweat.

4. Constant copious sweat day and night; in the daytime very slight sweating, especially from every mental effort; sweat night and morning with thirst; cold, sticky sweat, often sour or offensive smelling.

**Hyoscyamus.**

1. Pulse accelerated, full, hard, and strong; more seldom slow and intermitting; great swelling of the veins.

2. Chill and shuddering over the whole body, and heat of the face, ascending from the feet; nocturnal coldness, arising from the sacro-lumbar region to the back; he cannot get warm in the bed at night; universal coldness of the body, with red, hot cheeks, or chill alternating with heat.

3. Burning heat over the whole body, every evening; with the heat uncommon rush of blood to the head, with putrid taste in the mouth.

4. Continued, debilitating sweat in the sleep; many and very copious sweatings; cold, and sometimes sour-smelling sweats; sweat most on the legs.

**Ignatia.**

1. Pulse generally hard, full, and rapid, with throbbing of the veins; more seldom small or slow; in other particulars very changeable.

2. Chill and coldness, with increase of pains; chill always with thirst, and relieved by external warmth; chill, often only on the posterior portion of the body; external coldness with internal heat; internal chill with external heat.

3. Merely external heat without thirst, with intolerance of external warmth; external heat with redness and internal shuddering chill; attacks of flying, external heat; constant rapid changes of heat and coldness; one-sided burning heat of the face.

4. Slight sweating, often only on the face; sensation as if sweat would break out, which does not; sweat while eating. The sweat is sometimes cold, but generally warm, and somewhat sour smelling.

**Iodine.**

1. Pulse large, hard, and accelerated, with great ebullition of blood and throbbing in the veins; pulse rapid but weak and thready. The pulse becomes more rapid from every movement.

2. Chill often alternates with heat; cold feet the whole night; chill with shaking also in a warm room.

3. Universal flying heat over the whole body; internal, dry heat, with coldness of the skin.

4. Very copious sweat at night; very debilitating sweat in the morning hours, sour-smelling, with great thirst.

**Ipecacuanha.**

1. Pulse greatly accelerated, but often imperceptible.

2. Chill generally of short duration, and soon passes into heat; internal chill, as if under the skin, increased by warmth; chill with thirst; thirst, coldness of the hands and feet; chill mostly with thirst.

3. Universal, continued heat, with dry parchment-like skin, after a short chill; evening, dry, anxious heat; sudden attacks of general heat, with cold hands and feet. The heat is mostly without thirst.

4. Very great sweat, mostly at night; biting, mostly sour-smelling sweat, often also cold; in a room frequent attacks of hot sweat.

**Kali-carbonicum.**

1. Pulse very various; often weak and slow, also often remarkably rapid and hard; sometimes the pulse is quicker in the morning, and slower in the evening; seldom the reverse; strong throbbing in the blood-vessels.

2. Chill mostly in the evening; through the day, at times, running, cold shuddering; coldness in the evening in a warm room, which ceases soon after lying down; after the pains chill follows very often.

3. Heat in the morning in bed; internal heat with external shuddering.

4. Sweat every night; morning sweat; easy sweating in the daytime from bodily movement and mental exertion; sweat on the upper part of the body especially, also increased by warm drinks; offensive or sour-smelling sweat; transpiration wanting, with inability to sweat.

**Lachesis.**

1. Pulse small and weak, but accelerated, often alternating with a full, strong beat, chiefly very irregular and intermitting.

2. Universal chill, with chattering of the teeth, longing for warmth, and external benumbing chill; shuddering chill running up the back, often every other day; chill and heat alternating, and from one place to another ; chill returning every other day.

3. Heat evenings, especially on the hands and feet; evenings and nights burning of palms of the hands and soles of the feet, or nocturnal heat, as if from ebullition of blood, with great sensitiveness of the external throat; internal sensation of heat with cold feet.

4. Copious sweat with most of the ailments; great inclination to sweating; cold sweat, or bloody, or staining yellow or red.

**Laurocerasus.**

1. Pulse extremely irregular, sometimes small and slow, often imperceptible; again somewhat accelerated; seldom full and hard.

2. Chill, and cold shuddering afternoons and evenings, not relieved by external warmth; alternating chill and heat; want of natural bodily warmth.

3. Heat after the chill, evenings, till midnight; heat running down the back.

4. Sweat mostly with the heat, and still after this continuing till toward morning ; sweat after eating.

**Ledum.**

1. Pulse full and quick. The pulse is often perceptible on one side, while it is not on the other.

2. Chill with shuddering or thirst, long-continued, with sensation as if some parts were having cold water poured over them; coldness, and want of life warmth; mornings and forenoons predominant chill with thirst; universal chill, with heat and redness of the face.

3. Heat without thirst prevailing toward evening; evening, burning of the hands and feet; heat alternating with sweat.

4. Sweat the whole night, with disposition to uncovering: offensive or sour-smelling night-sweat; sweat from the least movement, most on the forehead; itching sweat.

**Lycopodium.**

1. Pulse somewhat accelerated, only in the evening, and after eating; evening, ebullition of blood with restlessness and trembling; sensation as if the blood were stopped.

2. Chill afternoon and evening (from four to eight o'clock), with dead hands and feet; chill in the evening which hinders sleep; one-sided chill, mostly the left; chill and then sweat without previous heat; chill and heat alternating; want of natural bodily warmth.

3. Burning heat over the whole body, mostly toward evening, with frequent drinking, but little at a time, with much urine and constipation; heat of one foot (left), and coldness of the other (right).

4. Sweat in the daytime from the least movement, most on the face; night and morning sweat, often with coldness of the face; sticky night-sweat; the sweat is often cold, sour, or offensive smelling, or with smell of blood or onions.

**Magnesia-carb.**

1. Pulse somewhat accelerated, only at night.

2. Chill and shuddering, with external coldness, evenings, and after lying down, only slowly disappearing; chill running down the back, seldom ascending from the feet.

3. Heat mostly in the forenoon, often with sweat only on the head; heat in the evening after the chill; nocturnal, anxious, and internal heat, with restlessness and aversion to uncovering.

4. Sweat the whole night, most in the morning hours; the sweat is fatty, staining the linen yellow, and sour or offensive smelling.

**Magnesia-muriatica.**

1. Pulse somewhat accelerated, with ebullition of blood while sitting.

2. Chill in the evening between four and eight o'clock, even by a warm stove, slowly disappearing after lying down.

3. Heat after the chill from evening till midnight; evening heat with sweat only on the head.

4. Sweat with thirst from midnight till morning; morning sweat.

**Menyanthes-trifoliata.**

1. Pulse slow with the chill, with the heat accelerated.

2. Chill predominant; chill with shuddering over the back; ice-cold hands and feet, and cold sensation in the abdomen; general chill, which disappears by a warm stove, except on the back ; running shuddering without chill (as if from listening to a terrifying story), only on the upper part of the body ; chill sensation on the fingers and legs.

3. General heat in the evening, most on the head, with cold feet, and sensation of heat, especially in the back, mixed with sensation of chill, especially in the abdomen.

4. Sweat in the evening in bed immediately after lying down, often continuing the whole night.

**Mercurius-vivus.**

1. Pulse irregular, mostly full and accelerated, with strong throbbing of the blood-vessels, sometimes weak, slow, and trembling, seldom intermittent; disappearing pulse with warmth of the body ; ebullition of the blood, with trembling from the least exertion.

2. Chill in the morning while rising, but most in the evening when lying down, as if cold water were poured over one, and not relieved by the warm stove ; nocturnal chill with frequent urination ; chill alternating with heat ; often only on single parts ; internal chill, with heat of the face.

3. Heat in the bed, and chill out of it ; heat after midnight with great thirst for cold drinks ; anxious heat, with pressing together of the chest, alternating with chill.

4. Sweat toward morning, with thirst and palpitation of the heart ; great sweating from the least exertion, even from eating ; sweat in bed, evenings, before going to sleep ; copious night-sweating ; very debilitating sweats ; sour or offensive smelling sweats, also cold, fatty, or sticky, and burning on the skin ; with almost all pains sweating, or at least dampness of the skin.

**Mercurius-corrosivus.**

1. Pulse small, weak, and often intermitting ; sometimes trembling.

2. Chill from the least movement, and, in the open air, almost constantly with cuttings in the abdomen ; evening chilliness, especially on the head ; chill at night in bed.

3. External heat with yellow skin ; burning and pricking heat in the skin ; heat while stooping, and chill while rising up.

4. Night-sweat; toward morning the sweat becomes offensive; cold sweat only on the forehead; the whole skin is covered with cold, anxious sweat.

**Mezereum.**

1. Pulse full, hard, accelerated in the evening, sometimes intermittent.

2. Chill predominates even in the warm room; chill with external coldness and thirst for cold water, without desire for warmth; constant thirst with the chill, with dryness of the back part of the mouth or accumulation of saliva in the fore-part, but without desire for drink, chilliness and shuddering with almost all ailments; great sensitiveness to cold air; chill which runs from the upper arms to the back and down to the feet.

3. Heat in bed, most on the head; internal heat with external chill.

4. Sweat in sleep, immediately after the chill, without precedent heat.

**Moschus.**

1. Pulse very full and accelerated, with strong ebullitions of blood; great anema with weak pulse and fainting.

2. Chill and shuddering which spreads itself from the scalp over the whole body; sensation as of a current of cold air on uncovered parts; external coldness with internal heat; shuddering, alternating with heat; one cheek hot without redness, the other red without heat.

3. Burning heat evenings in bed, often only on the right side, with restlessness and disposition to uncovering; one hand burning hot (and pale), the other cold (and red.)

4. Sticky sweat which smells like musk, in the morning.

**Muriatic Acid.**

1. Pulse weak and slow and intermitting every third beat.

2. Chill predominates; evening chill with cold sensation on the back, with external warmth and burning of the face; shuddering over the whole body with hot cheeks and cold hands; chill and heat without thirst.

3. Internal heat with disposition to uncovering and restlessness of the whole body; burning heat, especially at the palms of the hands and soles of the feet.

4. Sweat in the first sleep, till midnight, especially on the head and back; night and morning sweat; evening in bed the sweat on the feet is cold at the beginning.

**Natrum-carb.**

1. Pulse, in the night, most excited, with ebullition of blood through the whole body.

2. Chill and internal chilliness with shuddering, the whole day, most in the forenoon, with cold hands and feet, and hot head, or the reverse, with warm hands and cold cheeks; evening chilliness with dull confusion (*eingenommenheit*) of the head, followed by heat, with sleep.

3. Heat with weakness and sleep (without headache, which, with Natrum-mur., is very severe); heat running from the neck downward to the back, and disturbed temper; heat with concomitant sweat over the whole body.

4. Copious, anxious sweat from the least movement; burning on the forehead where the hat touches; copious night-sweats; night-sweat alternating with dryness of the skin; cold, anxious sweat with the pains.

**Natrum-muriaticum.**

1. Pulse extremely irregular, often intermittent, especially if lying on the left side, with throbbing and swelling of the blood-vessels; pulse now quick and weak, and now full and slow; the pulse-beat perceptibly shakes the whole body.

2. Chill predominating, mostly internal, as if from deficient bodily warmth, with icy-cold hands and feet, most in the evening; long-continued chill from morning to noon.

3. Burning heat with the severest headache, often with shuddering on the back, and sweat on the epigastrium and soles of the feet; long-continued heat afternoons, with the severest headache, with inability to think, which gradually disappears in the subsequent sweating; with the heat there is mostly great thirst.

4. Copious sweating in which most of the ailments which appear with the fever cease; much sweating during the day and great disposition to this from every movement; night and morning-sweats; debilitating, somewhat sour-smelling sweat.

**Nitric Acid.**

1. Pulse uncommonly irregular; after one normal beat often follow two small and quick, and the fourth intermits; alternating hard, quick, and small beats.

2. Chill most afternoons and evenings, as well as after lying down; chill with concomitant internal heat; chill in the morning in bed, often preceding heat; constant chilliness.

3. Heat, especially on the face and hands; burning heat with sweating hands; nocturnal, internal, dry heat with inclination to uncovering; after eating, heat with sweat and great weakness.

4. Sweat every night, or every other night, most on the side on which one lies; sour, or offensive, or like horse-urine smelling sweat.

**Nux-moschata.**

1. Pulse somewhat quickened, as if from ebullition of blood.

2. Chill from every uncovering, and coldness in the open air, especially in damp, cold air, with great paleness of the face, immediately disappearing in a warm room; cold sensation on the feet with heat of the hands; chilliness in the evening with great sleepiness; chill and coma predominant.

3. Heat of the face and hands forenoons, with hypochondriac disposition, thirstlessness, and dryness of the mouth and throat.

4. Slight sweating, which sometimes is red like blood.

**Nux-vomica.**

1. Pulse full, hard, and quickened, especially during the heat; pulse small and quick, the fourth or fifth beat intermitting; pulse imperceptible.

2. Chill and coldness not relieved by extreme warmth; chill and shuddering, evening and night in bed till morning, increased by every movement and by drinking; chill with heat of the face; chill alternating with heat; chill and shuddering while moving in the cold, open air; sleep between chill and heat.

3. Universal, internal, burning heat; nocturnal heat without thirst; heat greatly aggravated from the least exertion or movement, also in the open air; heat with aversion to uncovering, and at the same time chill; heat with disposition to uncovering, from which appear other ailments; heat before the chill; heat of single parts with chill and shuddering in others; heat which is as if streaming from the throat.

4. Sweat after midnight and mornings; sour or offensive sweat; sweat one-sided or only on the upper part of the body; cold, sticky sweat on the face; sweat with relief, especially of the pains of the limbs.

**Opium.**

1. Pulse very various, full and slow; with difficult morning respiration; quick and hard with heat and rapid, anxious respiration; toward the end weak and intermitting.

2. Chill and diminished bodily warmth with benumbing and weak, hardly-perceptible pulse; the whole body is stiff and cold; coldness only on the limbs.

3. Heat with sweating skin predominates, extending itself from the head or stomach over the whole body; burning heat of the whole sweating body, with great redness of the face, and subsequent snoring sleep; heat with disposition to uncovering.

4. Copious sweat over the whole, burning, hot body, with snoring sleep; in the morning general, copious sweating with disposition to uncovering; sweat on the upper part of the body, with dry heat of the lower part; cold sweat on the forehead.

**Paris-quadrifolia.**

1. Pulse full, but slow.

2. Chill most toward evening, with internal trembling; one-sided chill, right, with warmth of the other side; during the chill sense of contraction of the skin and all parts of the body; chilliness with goose-flesh; nights, in bed almost constant cold feet.

3. Heat from the neck down to the back; heat with sweat on the upper part of the body.

4. Sweat in the morning on waking, with biting itching.

**Petroleum.**

1. Pulse, from every movement, is made stronger, full, and accelerated; in repose immediately becomes slow.

2. Chill mostly toward evening, earlier or later; chilliness through the whole body with subsequent severe itching; in the evening, internal chill and heat at the same time; chilliness in the open air; chill with headache and excessive coldness of face and hands.

3. Heat in the evening after a chill, with cold feet; heat after midnight and mornings, in bed; flying heat over the whole body, in repeated attacks in the day-time (six to ten); sensations of heat over the whole body, and great burning of the skin.

4. Copious sweat every night; slight sweating, especially on the forearms and legs; sweat immediately after the chill without preceding heat.

**Phosphorus.**

1. Pulse various; generally quickened, at the same time full and hard, but sometimes weak and small; more seldom slow and intermitting; strong ebullition of blood and throbbing of the carotids.

2. Chill almost only in the evening, without thirst, with aversion to every uncovering, and greatly swollen veins of the hands; internal chill with shuddering, not relieved by the warmth of the stove; chill and heat alternating at night; chilliness in the evening till midnight, with great weakness and sleep; nocturnal chill with diarrhœa; chill running down the back.

3. Flying heat over the whole body, but first on the hands; universal, anxious heat afternoons and evenings, with burning on the hands and face; nocturnal heat which prevents sleep, mostly after midnight; heat rising up the back; heat with coma.

4. Sweat, most on the head, hands, and feet, with copious urine; sweat only on the forepart of the body; after midnight and in the morning copious sweating followed by great weakness; sticky sweat; the transpiration often smells like sulphur.

**Phosphoric Acid.**

1. Pulse irregular, sometimes intermitting one or two beats, mostly small, weak, but quick, but often full and strong; great ebullition of blood, with great restlessness; swollen veins.

2. Chill with shuddering and shaking; generally evenings; chill and heat alternating in frequent attacks; one-sided cold sensation on the face; during the chill an especial sensitive cold sensation in the ends of the fingers and in the abdomen.

3. Internal, dry heat, without thirst, and without complaints, at all times of the day; general heat with loss of consciousness and coma; anxious heat in the evening, with strong ebullition of blood; heat of head with cold feet.

4. Sweat most on the occiput and neck while sleeping in the daytime; copious night and morning sweatings, with anxiety; uncommon disposition to sweating day and night; sticky sweat.

**Platina.**

1. Pulse small and weak, and often trembling.

2. Chill in the evening with trembling, and sensation of trembling through the whole body; shaking chill in passing from a room into the open (even warm) air; chill and chilliness predominates, with pettishness, which passes off later in the heat; alternations of chill and symptoms of intellect and disposition.

3. Heat with sensation of burning redness of face when these are neither noticeable or present; flying heat mingled with shuddering; gradually increasing and gradually diminishing heat.

4. Sweat only with sleep, which disappears immediately on waking.

**Plumbum.**

1. Pulse very various and irregular; mostly small and contracted and slow; sometimes hard and slow; sometimes also small and quickened; seldom full and feverish or intermittent.

2. Chill predominates, which increases toward evening, with great thirst and redness of the face; in the evening internal chill with external heat; chilliness in all the limbs; coldness in the open air and from motion.

3. Heat with thirst, anxiety, redness of the face, and sleepiness; evenings and nights internal heat, with yellowness of the whole inner mouth.

4. Sweat anxious, cold, or sticky.

**Pulsatilla.**

1. Pulse weak and small, often hardly perceptible, but quickened; seldom slow; evenings throbbing in the blood-vessels; swollen veins in the evening heat.

2. Chill, coldness and shuddering predominate; constant internal chilliness, even in a warm room; chill increased toward evening; chill with the pains; chilliness with overrunning heat; one-sided coldness with sensation of numbness; in the evening cold drawings through the back; evenings and before midnight constant running chill without shuddering; thirst before the chill and before the heat, seldom with either.

3. Heat after the chill, with anxiety and redness of the face; general internal, dry heat, without external heat, evenings or nights; heat of the face or of one hand, with coldness of the other; heat of the body with coldness of the extremities; attacks of anxious heat as if hot water were poured over one.

4. Copious sweat in the night or morning; sweat during sleep; soon disappears on waking; easy sweating in the daytime; one-sided sweat, sometimes only on the face and hairy scalp; night-sweat with benumbing coma; sweat often smells sweetish, sour, or moldy, or like musk, and is sometimes cold.

**Ranunculus-bulbosus.**

1. Pulse evenings hard and full and quick; mornings slow.

2. Chill predominant, with heat of the face, mostly afternoons and evenings; after the (noon) meal chilliness, with heat of the face; in the open air he chills, most external, on the covered chest; the fever often consists merely of chill.

3. Heat in the evening, especially on the face, often only one-sided (right), with cold hands (and feet); heat with co-existent internal chill.

4. Sweat but little, and only in the morning on waking.

**Ranunculus-sceleratus.**

1. Pulse quick, full but weak, with the nocturnal heat.

2. Chill and chilliness while eating.

3. Heat in the evening in a room, after going in the open air; nocturnal, dry heat, with great thirst, and strong ebullition of blood, most after midnight; the heat is predominant.

4. Nocturnal sweat after the heat toward morning, most on the forehead.

**Rheum.**

1. Pulse only a little quickened.

2. Chill alternating with heat; one cheek red the other pale; internal shuddering with external warmth.

3. Heat over the whole body, most on the hands and feet, with cold face; heat preponderates.

4. Sweat from the least movement; cold sweat about the mouth and nose; sweat on the forehead and scalp; the sweat is yellow and smells like rhubarb.

**Rhododendron.**

1. Pulse weak and slow.

2. Chill over the whole body mornings, in bed, and in day while cold air blows on him; chill alternating with heat; in the evening and often lying down, icy-cold feet for long time in bed.

3. Heat in the evening, with cold feet; sensation of warmth in the hands, although they are cold to touch; evenings feverish heat, with burning of the face.

4. Copious, debilitating sweat, especially while moving in the open air; offensive sweat on the arm-pits; aromatic-smelling sweat; while sweating itching and crawling on the skin as if from insects.

**Rhus-toxicodendron.**

1. Pulse irregular, generally accelerated, but weak, languid, and soft, sometimes imperceptible and intermitting.

2. Chill most frequently in the evening, often going from the feet or the shoulder-blades; chill as if cold water were poured over him, or as if the blood ran cold through the veins; cold sensation from every movement; chill with increased pains, especially in the limbs; chill with heat and redness of the face; chill and heat in rapid alternation; one-sided chill; coldness of the right side with heat of the left; coldness of the head and back side of the body, with heat of the forepart; coldness and paleness of the face, alternating with heat and redness.

3. Heat after the chill, often with concomitant sweating with relief of accompanying symptoms and pains in the limbs; universal heat as from pouring hot water over one, or as if the blood ran hot through the veins; flying heat with sweat going from the umbilicus, often alternating with chill; heat with nettle rash.

4. Universal sweating, mostly during the heat, often excluding the face; copious night and morning sweats; moldy, offensive, or sour smelling sweat. Sweat with the pains while sitting; while sweating, severe itching of the eruption.

**Ruta Graveolens.**

1. Pulse somewhat quickened during the heat.

2. Internal chill, with shaking and shuddering, even by a warm stove. Running chill over one side of the head. Chill mostly on the back, which runs upward and downward. Chill, with heat of the face and great thirst.

3. Heat over the whole body; most in the afternoon, without thirst, but with anxiety, restlessness, and oppressed breathing; external and internal heat of the face with red cheeks, and cold hands and feet; frequent sudden attacks of flashes of heat.

4. Cold sweat on the face in the morning in bed; general sweat after going in the open air.

**Sabadilla.**

1. Pulse small but somewhat jerking. Great ebullition of blood and throbbing of vessels. Sensation of stagnation of the blood.

2. Chill afternoons or evenings, returning exactly at the same hour, often without subsequent heat. Chill predominating, especially on the extremities, with heat of the face. The shuddering chill constantly from below upward. The chill is relieved by the warmth of the stove.

3. Heat, most on the head and face, often interrupted by shuddering chill, constantly recurring at the same hour, thirst only between the chill and heat. Sweat often with the heat. Nights and mornings internal heat.

4. Sweat mornings in sleep. Hot sweat on the face with cold on all the rest of the body.

**Sabina.**

1. Pulse irregular, mostly quick, strong, and tense. Strong throbbing of the vessels of the whole body.

2. Chill in the evening, with repeated cold shudderings. Great coldness through the day. Shuddering with darkness before the eyes, and subsequent sleepiness. Cold sensation in the whole (right) leg.

3. Insupportable, burning heat of the whole body, with great restlessness. Flashing heat of the face, with chill of all the rest of the body and cold hands and feet.

4. Sweat every night.

**Sambucus.**

1. Pulse various; mostly small and very quick; sometimes intermitting; often also full and slow. Great ebullition of blood in the body.

2. Chill running over the whole body, with crawling here and there. Shuddering chill with very cold hands and feet.

3. Dry heat over the whole body as soon as he sleeps, after lying down, with aversion to uncovering, without thirst. Burning heat of the face, with cold feet.

4. Uncommonly copious sweating day and night, but only while awake, first breaking out on the face, and continuing even into the apyrexia. Extremely debilitating sweat. Universal night-sweat, except of the head, increased toward morning. Constant sweating while awake, which, during sleep, passes into dry heat.

**Sarsaparilla.**

1. Pulse somewhat quickened, with great ebullition of blood, mostly toward evening.

2. Chill predominant day and night. Frequent shuddering chills, most in the forenoon, running from below upward. Coldness of the whole body, most on the feet, except the face and chest, also by a warm stove. He is worse during the chills.

3. Heat in the evening, with ebullition of blood and palpitation of the heart. Evening sensation of warmth with increased sense of improvement.

4. Sweat evenings, with the heat, but only on the forehead.

**Scilla.**

1. Pulse small and slow, but somewhat hard.

2. Nocturnal internal chill, with external heat. Chilliness toward evening, while walking, not while sitting.

3. Dry, burning, internal heat of the whole body predominates, with cold hands and feet, and intolerance of uncovering; great sense of heat in the whole body afternoons and evenings, mostly with cold feet. Chill and pains from every uncovering during the heat.

4. Sweat wholly wanting. Absence of transpiration even with the greatest burning heat.

**Secale-cornutum.**

1. Pulse often unchanged, even in the severest attacks. Pulse mostly slow and contracted, sometimes intermitting or suppressed, only in the heat somewhat quickened.

2. Severe but brief chills, with internal, burning heat, soon following with great thirst. Unpleasant sensation of coldness in the back, in the abdomen and limbs.

3. Great, long-continued, dry heat, with great restlessness and great thirst.

4. Sweat, especially on the upper part of the body. Universal, cold, sticky sweat.

**Selenium.**

1. Pulse but little quickened, even with great ebullition of blood.

2. Constant chill, alternating with heat.

3. Mostly external heat, like burning in the skin, and only on single parts.

4. Very great sweat, especially on the chest, in the arm-pits, and on the genitals; sweat from the least movement; sweat in every sleep, both day and night; excessively easy sweating; the sweat makes a yellowish or white stiff spot on the linen.

**Polygala-senega.**

1. Pulse irregular, mostly hard and quickened, with great blood ebullition; seldom soft.

2. Chill and chilliness, almost only in the open air, with weakness of the legs and oppressed respiration; shuddering over the back, with heat of the face, chest affections, and other sufferings.

3. Only rapid flashes of heat.

4. Sweat wholly wanting, and appears only in the secondary effects of the drug.

**Sepia.**

1. Pulse at night rapid and full, and then often intermitting; in the day, slow. The pulse is especially accelerated by anger and motion; great ebullition of blood and throbbing of the vessels.

2. Chill often appears first after previous heat; chilliness in the evening in the open air, and from every movement; chill, alternating with heat; more thirst with the chill than with the heat; shuddering chill with the pains; deficient animal heat.

3. Attacks of flying heat in the daytime, especially afternoons and evenings, as well while sitting as when going in the open air, and from mental excitement, mostly with thirst and redness of the face; attacks of heat, as from hot water poured over one.

4. Sweat copious, more after than during movement; continued debilitating sweat; constant night and morning sweating; sweat only on the upper part of the body; sweat, anxious, offensive, sour, or smelling like elder blossoms.

**Silicea.**

1. Pulse small, but hard and quick; often irregular, and then sometimes slow; the blood is very easily in ebullition.

2. Strong chill evening in bed, increased by every uncovering; great chilliness, especially from every movement; constant internal chilliness and want of animal heat.

3. Heat predominant; frequent short attacks of flying heat in the daytime, most on the face; universal heat, with great thirst afternoons, evenings, or through the whole night; in the daytime, typical recurring heat, without previous chill, followed by slight sweat.

4. Debilitating sweat in the night or only in the morning; sweat from moderate movements, most on the head and face; great sweating only on the head; nocturnal sour or offensive sweat; sweat wholly wanting.

**Spigelia.**

1. Pulse irregular, mostly strong and slow ; pulse trembling ; pulse quicker in the evening, slower in the morning.

2. Chill in the morning, often recurring at the same hour ; chill, alternating with heat or sweat ; chill of single parts with warmth of others ; universal running chill, with concomitant heat ; chilliness from the least movement ; the chill proceeds from the chest.

3. Heat, especially on the back ; nocturnal flying heat, with thirst for beer, and heat of face and hands with chill on the back.

4. Nocturnal, offensive sweat, with concomitant heat ; sticky sweat on the hands ; cold sweat.

**Spongia-tosta.**

1. Pulse very quick, full, and hard ; strong ebullition of blood and swelling of the veins.

2. Chill, with shaking, even by a warm stove, most on the back.

3. Great heat, soon after the chill, with dry, burning skin over the whole body, with exception of the thighs, which remain cold, numb, and chilly ; attacks of overrunning, flying heat ; anxious heat, with red cheeks and weeping and inconsolable disposition.

4. Evenings, cool sweat on the face ; morning, sweat over the whole body.

**Stannum.**

1. Pulse small and quick.

2. Evening chill, especially over the back, often preceding heat, with sweat ; shuddering chill daily about ten o'clock forenoon ; chill mostly on the head ; with slight chill, strong chattering of the teeth, as if from convulsion of the masticatory muscles ; in a shuddering chill, in the forenoon, great sensation of numbness in the ends of the fingers.

3. Heat in the afternoon (from four to five o'clock), recurring daily, with concomitant sweat ; burning heat in the limbs every evening, most on the hands ; anxious heat, as if sweat would break out, in repeated attacks ; anxious sensation of heat from the least movement; predominant internal sensation of heat.

4. Very debilitating sweat nights and in the morning, most on the throat; very debilitating universal sweat from the least movement; damp or moldy-smelling sweat.

**Staphisagria.**

1. Pulse very quick, but small and often trembling.

2. Chill and coldness predominate; chill and coldness in the evening, often without subsequent heat; evenings, severe chill, with shaking and shuddering and heat of the face; shuddering chill afternoons (three o'clock), from movement in the open air relieved; chill from the back up over the head, even by a warm stove; chill running down the back.

3. External heat, with thirst after midnight, followed by chill toward morning; nocturnal, burning heat, with disposition to uncovering, especially of the hands and feet.

4. Copious sweat and great inclination to sweating (inability to sweat, with headache and paleness of the face). Nocturnal sweat, smelling like spoiled eggs. Cold sweat on the forehead and on the feet.

**Stramonium.**

1. Pulse extremely irregular, mostly full, hard, and accelerated; then, again, small and quick; sometimes slow and hardly perceptible; also intermittent and trembling.

2. Chill and universal coldness, with redness of the face and jerking, often very long continued. Universal coldness afternoons after previous heat of the head and face, and subsequent universal heat. During the chill uncommon sensibility to uncovering. Chill running down the back.

3. Heat of the whole body, with vivid redness of the face, mostly with concomitant sweat; hot, red face, with cold hands and feet; anxious heat with vomiting.

4. Copious sweat, even with the heat, over the whole body, with great thirst; oily, offensive sweat; universal cold sweat.

**Strontium.**

1. Pulse full and hard, with strong throbbing in the vessels.

2. Chill in the forenoon, running down from the back to the back part of the thighs. Shuddering chill over the head and shoulder-blades.

3. Nocturnal dry heat, with thirst. Heat which streams from the nose and mouth.

4. Sweat in the morning. Nocturnal sweat, mostly on the painful part, with increase of the pain from uncovering.

**Sulphur.**

1. Pulse full, hard, and somewhat quickened, sometimes intermitting.

2. Chill and chilliness, mostly internal and without thirst, mostly evenings, but also at other times of the day. External chill with concomitant internal heat and redness of the face. Severe chill at night in bed. Chills in the forenoon, in the afternoon heat, with cold feet. Chill with thirst, after previous heat. Chill going out from the toes. Chill running up the back.

3. Afternoons and evenings heat, with dry skin and great thirst. Frequent attacks of flying heat. Great heat at night, without thirst, often preceding chill with thirst.

4. Sweat nights and mornings. Copious sour smelling sweat the whole night. Evening sweat, most on the hands. Copious sweat from the least movement. Anxious, debilitating sweat, empyreumatic, sour-smelling, seldom offensive, sometimes also cold sweat. Night-sweat, only on the nape of the neck and occiput.

**Sulphuric-acid.**

1. Pulse small and weak, but quick.

2. Chill in the daytime, mostly in a room, relieved by movement in the open air. Frequent shudderings running down the body.

3. Heat in the evening, also after lying down in bed. Frequent flashes of heat in the evening, especially after movement. Attacks of flashing heat with concomitant sweat (in the climacteric period).

4. Copious sweat, most on the upper part of the body. Copious sweating on the feet. Sweat from every movement, which continues long after sitting down; sour sweat; cold sweat immediately after eating warm food.

**Taraxicum.**

1. Pulse?

2. Chill and chilliness, especially after eating and drinking. General chill, with headache. Shuddering chill in the open air.

3. Heat at night on waking, especially on the face and hands.

4. Very copious sweat the whole night, most in the first sleep before midnight. Greatly debilitating sweat, which causes smarting of the skin.

**Thuja-occidentalis.**

1. Pulse slow and weak, in the morning; in the evening quick and full. Strong throbbing in the veins evenings. Great swelling of the veins.

2. Attacks of chill at different times of the day, most toward evening. Chill on the left side of the body, which is cold to the touch. After midnight and morning chill without thirst. Internal chill with external heat and great thirst.

3. Heat evenings, especially of the face. Burning of the face without redness. Dry heat of covered parts.

4. Sweat at the beginning of sleep. Sweat of the uncovered parts of the body, with dry heat of the covered, and the reverse. Anxious, sometimes cold sweat. Sweat immediately after the chill without heat. The sweat is often fatty, sometimes offensive, or sweetish smelling, like honey.

**Valeriana-officinalis.**

1. Pulse very irregular and unequal, mostly very quick and somewhat tense, but also at times small and weak.

2. Brief attacks of chill which soon pass into protracted heat. The shuddering chills start mostly from the neck and run down the back.

3. Predominant, long-continued, and universal heat, often with sweat on the face. Flashing heat of the face. Increased heat evenings and when eating. Predominant heat with thirst.

4. Copious sweat, especially nights, and from movement, with great continued heat. Frequent, sudden attacks of sweating, especially on the face and forehead, which disappear equally sudden.

**Veratrum-album.**

1. Pulse irregular, most frequently small and thready, weak and slow, often lost and wholly imperceptible, seldom hard and quick. The blood runs like cold water through the veins.

2. Chill and coldness, mostly external, with internal heat, and cold, sticky sweat. Shuddering chill, with sweat, which, with the sweat, passes into universal chill. Predominant chill and coldness, running from above downward. Chill alternating with heat. Chill increased by drinking. Icy coldness of the whole body.

3. Almost only internal heat with thirst, without desire to drink. Heat evenings, with sweat. Heat rapidly alternating with chill. Alternating chill here and there on single parts.

4. Copious sweats mornings and evenings, or through the whole night, as well as with every stool. Cold, sticky, sour, or offensive, sometimes bitter-smelling or yellow-staining sweat, constantly with deadly paleness of the face. Cold sweat over the whole body, most on the forehead. Easy sweating in the daytime from every movement.

**Vitex Agnus Castus.**

1. Pulse weak and slow, often imperceptible.

2. Internal chill with trembling; with external warmth of the skin, chill alternating with heat; great chilliness, with cold hands; chill predominates.

3. Overrunning, burning heat, mostly on the face, with cold knees, evenings, in bed.

4. Sweat, mostly on the hands, while going in the open air.

**Zincum.**

1. Pulse, evenings, small and quick; in the morning and daytime slower; pulse sometimes intermittent; great throbbing of blood-vessels during the heat.

2. Chill, mostly beginning after eating, and continuing till late in the evening, and even in bed; shuddering chill in the open air, and from touching a cold object; frequent alternations of chill and heat in the daytime; shuddering chill, which runs down the back; shuddering chill before an approaching storm; constant external chilliness with increased internal warmth.

3. Internal heat with cold sensation in the abdomen and feet; anxious sensation of heat, without external heat, through the night; heat of the face with cool body, forenoons; flashes of flying heat with great trembling and short, hot breath.

4. Copious sweat through the night with inclination to uncovering; very slight sweating through the day, from movement; offensive sweat.

The above groups of symptoms produced by the drugs named are far from being the whole number recorded as having resulted from the provings which have given us our materia medica, by which resemblances to the elements of this fever have been disclosed, thus exposing the relationship of curatives to this troublesome disease. Indeed some of the most important remedies for the fever are not found named here, the object being, not to present a complete view of our armamentarium for the conquest of this fever, but, as has been said, to give a sufficient number of these groups for such a comparative study of this element of drug action as will enable one, after familiarizing himself with these and with their differentiations, the more readily to recognize the similar differentiations in examples of disease he may be called to treat, in which differentiations the relationship of curatives to diseases only is found. If many of these seem, on a cursory perusal, to be but little other than in each a repetition of the facts of the other, a careful study will bring out the differences which characterize each, and by which each is related to the case, the specific for which it thus stands declared. If, to the unpracticed, these sometimes may seem to be trifles, let him remember that in pathogenesis and symptomatology *there are no such things as trifles.* Symptoms which to the diagnostician may be but of trifling import are often decisive in choice of the specific.

Not only are the drugs represented in the groups of symptoms given above not all of those related to intermittent fever by similar symptoms, but elements of their own action, or hints from these more or less decisive in the choice of them, for cases in which they may be found the specifics are omitted. We shall add a few of these hints or helps to right selection of remedies in cases for which we have to care.

We will, in addition to what we have already said of *Aconite,*

remark that it will seldom be found curative in this fever, in cases where the morale is tranquil. It is here, as in most sicknesses for which it is the appropriate remedy, that loud complainings and restless, fearing, anxious morale is met. The intolerance of the bed-covering during the heat, which it manifests and shares with some other remedies, is a reason for its selection of some weight, if the other symptoms of *Aconite* are present, especially its peculiar morale—*i. e.*, where the symptoms of a case are so found in two or more drugs as to put the prescriber in doubt which he shall choose, this intolerance may be a deciding symptom. The same may be said of "sweat mostly on parts covered."

Of *Apis*, it may be noted, its chilliness with heat of hands and feet, in this being nearly the opposite of *Belladonna*, which has cold hands and feet, with hot head and face.

*Arnica.*—In addition to the symptoms of this drug given, which are quite characteristic and remarkable, it may not be amiss to say that in the beginning of the treatment of a case which has been unsuccessfully treated with abusing doses of Quinine, and notwithstanding the *quasi* old school "bragging" of success with this drug (recently mixed in the description of the treatment of this fever by pretending homœopathists, who should have known better), there are many such met. *Arnica* is often the very best remedy with which to begin to remedy the evils of this so unscientific and mischievous practice. It has brought prompt relief and made a really scientific (*i. e.*, a homœopathic) cure possible and easy by properly-selected homœopathic remedies, often this antidote to Quinine.

*Arsenicum*, perhaps after Quinine, the most frequently-administered remedy for this fever, both by old school and new, is no more a universal specific for the disease than is its near relative *Cinchona*. It is worthy of remark, and a fact of no little interest, that though these two drugs are so similar in many of their recorded effects on the organism, in those by which they are homœopathically related to this fever, they are so nearly exact opposites. The one notable fact in the elements of arsenical action which are in relation to the therapeutics of this fever is the indefiniteness and irregularity of their expression ; and often one of the paroxysmal elements is wanting altogether. The chill and heat may either or both be slight, mixed, *i. e.*, present at the same time, or alternating, in more or less rapid succession, or be wholly wanting in symmetry of proportion as to intensity and duration of action. With *Cinchona* the opposite is true. The paroxysmal elements are clearly expressed, the three being in symmetrical proportion each to the other as to intensity and duration, and each always present. *Cinchona* is rarely curative where the paroxysm is imperfectly expressed in either of its three constituent elements. The chill, heat, and sweating are each distinct. In the element of thirst, the two drugs have distinct characteristics. *Arsenic* has great thirst in all the stages of the paroxysm, but the patient drinks but little at a time, as if it were rather a sense of dryness of the mouth and throat than ordinary thirst. It has also an intense, inextinguishable thirst for *cold* drinks, which is not satisfied with the small quantity of liquid, as in the other form. In other cases the thirst is wanting throughout the paroxysm. The thirst of *Cinchona* is different and peculiar. It is present before or after the chill, and not with it, as also with the heat. It is before the chill, between this and the heat, and not with either, and is great *with the sweat*, and always for *cold* drinks. *Arsenic* is also often curative of cases complicated with the effects of the abuse of *Cinchona* or any of its constituent elements. If, with other similar symptoms of Arsenic there be its characteristic bodily restlessness, which does not permit rest in any one place or position, it may be given with expectation of curative results. If, on the other hand, there be bodily tranquillity, the remedy should not be given but for the strongest reasons—*i. e.*, many other and clearly-expressed symptoms of the drug.

*Belladonna* may be given with confidence if there be coldness of the extremities with heat of the body and face, and all the more if there be delirium with the heat. It is a reason for comparison of a case with this drug if the chill be initiated in and *proceeds from the arms*. It is to be borne in mind when *Belladonna* is examined, with reference to its administration in this fever, that most of its actions on the organism are characterized by violence. Those by which its relationship to this fever is declared are not exceptions to this general characteristic. It can be called for but seldom in cases where the morbid phenomena are but mildly expressed.

*Bryonia*, in its febrile phenomena, is characterized by violence of expression. The pulse is full, hard, quick, tense. The chill is likely to be predominant, accompanied by thirst. The heat is burning, as if the blood burned in the veins. The sweat is copious. It presents in its morale a double character, similating *Pulsatilla* in its despondency and fearfulness in the one part, and *Nux-vomica* in its proneness to petulance and anger, does not like to be disturbed, and is averse to movement. In this last it is differentiated from *Pulsatilla*, the pains of which are, for the most part, relieved by movement, and intensified by repose, especially by lying in bed.

*Cantharis* has two peculiarities which may readily indicate it to the prescriber. First, chill followed by thirst, without heat. Second, the urinous smell of the sweat.

*Capsicum* is a remedy often called for in the treatment of intermittents. It may be profitably studied in connection with *Carb-an.*, *Carb-veg.*, and *Ignatia.* The attack of each is mostly in the evening, and the chill is with thirst. The chill is predominant in each. I have no recollection of curing this fever with either *Caps.*, *Carbo-veg.*, or *Ignatia*, where the paroxysm was not initiated with chill, in the *evening*, with thirst. If the sweat follows the chill immediately, omitting the heat, in these circumstances, this will decide the choice for *Caps.* excluding its then allied associates. If the chill be relieved by external warmth, or the concomitant symptoms of the fever are relieved by eating, these facts will decide the choice for *Ignatia.* If the sweat be toward morning, or in the day-time from slight exertion, and if it colors the linen yellow, or if it be most on the thighs, it will indicate *Carbo-an.* If the chill be of the left side, or accompanied by great weakness, and there be many concomitant symptoms developed during the heat, as if the sweat smell sour or offensive, the choice will be *Carbo-*

*veg.* In the absence of these distinguishing characteristics of the different members of this group of remedies, in cases where the similar symptoms of it, as given above, leave the prescriber in doubt as to which is the true specific for his case, the decision of the choice must be determined by the concomitant symptoms of the case in hand. This rule applies to all cases where the *general* symptoms are so similar to those of two or more drugs as to leave the prescriber in doubt as to which he shall select.

*Causticum,* if the chill be predominant, with coldness of the whole *left* side of the body; or the chill be characterized by *internal* coldness and passes into the sweat without preceding heat. [See *Caps.*] Morning sweat at four o'clock.

*Chamomilla* is characterized by chill and shuddering of single parts of the body, while there is at the same time heat of other parts. Chill and coldness of the whole body with heat of *face* and *hot breath.* Heat and shuddering chill mixed, with one red and one pale cheek. Anxious heat with sweat on face and scalp. Sweat in sleep, most on the head and sour smelling.

*Chelidonium-majus.*—Chill internal, in the open air, which disappears in a room. Violent chill and coldness, most on hands and feet. [See *Menyanthes.*] Sweat in the night and morning, which disappears soon after waking.

China. [See *Arsenicum.*]

*Cicuta-virosa.*—The chill and coldness go from the chest and run over the extremities. Heat slight and only internal.

*Cina.*—Chill rises from the thighs to the head. Chill not relieved by external warmth. With canine hunger, nausea, clean tongue, vomiting, and diarrhœa. Vomiting of food first, then universal chill, then heat with great thirst. Heat with delirium.

*Clematis-erecta.*—Chill then sweat without preceding heat. [Compare with Bry., Caps., Caust., Dig., Lyc., Mez., Petr., Rhus-tox., Thuja, and Verat.]

*Cocculus.*—Constant chilliness with hot skin. Sweat on painful parts.

*Coffea-cruda.*—Chill increased by the *beginning* of every movement. Frequently recurring, internal shuddering chill with heat of the face or of the whole body. Sensation of chilliness with internal or external warmth. Dry heat at night with delirium. [See *Cina.*]

*Colocynth.*—Coldness of the hands or feet with warmth of the body. Chill and shuddering with the pains. Sweat at night with urinous smell. [See *Canth.*]

*Conium-maculatum.*—Shuddering first then heat with thirst and quick pulse, in frequent recurring attacks. Internal chill, wakes from sleep at five o'clock in the morning, with cold hands and soles of the feet, with hot face and great weakness after eight hours, with increased heat of face. Hot sensation of the whole body with perceptible warmth of the skin, dry, sticky lips, aversion to drinking, stringy saliva in the mouth, with aggravation from noises, bright light, and every movement, with inclination to sit with closed eyes.

*Crocus*, *Dulcamara*, and *Staphisagria* may be studied together. It will be found all have chill starting from the back. But *Crocus* and *Dulcamara* have thirst with the chill. *Staphisagria* has not. *Crocus* and *Staphisagria* have great thirst with the heat, while it is but slight with *Dulcamara*, or it has none at all. *Dulcamara* and *Staphisagria* have frequent urination during the chill, *Crocus* has not; *Dulcamara* has involuntary urination, and nausea during the chill, which neither of the others has. With *Crocus* the chill is aggravated by drinking and not with the others. With *Dulcamara* the chill is increased in a warm room, and not with the others. The attacks with *Staphisagria* are worse in the morning, *Crocus* in the afternoon, and *Dulcamara* in the evenings.

*For paroxysms* of the chill returning at the same hour.—Study together *Bovista*, *Hellebore*, *Kali-c.*, *Lycopodium*, *Sabadilla*, *Spigelia*, and *Thuja*. With *Bov.*, chill predominates, with thirst. It comes mornings or evenings, and nights; shuddering proceeding from the back; great coldness of hands and feet. *Hell.* has also predominant chill, with heat of the face; also alternating with pains in the joints. The shuddering proceeds from the arms. Chill in the daytime; heat as soon as one lies down in the evening or daytime, with concomitant sweat; aversion to drink during the heat. *Kali-carb.* Chill, mostly evenings. During the day shuddering chills run over the body; chills often follow the pains. Heat in the mornings in bed; internal heat with external shuddering, slight sweating in the daytime from motion or intellectual effort, or utter absence of sweat. *Lycopodium.* Chill afternoon and evening (from 4 to 8 P. M.), with dead hands and feet; chill one side (mostly left); chill followed immediately by sweat, without preceding heat. Heat of the left part, with coldness of the right; heat running over the whole body, mostly toward evening, with frequent drinking of small quantities; copious urine, and constipation of the bowels; copious sweat in the daytime from the slightest motion (see *Kali-c.*); most abundant on the face. *Sabadilla.* Chill afternoon and evening, often without subsequent heat; chill predominates, especially on the extremities, with heat of the face. The chill runs from below upwards; heat, if any, is frequently interrupted by intercurrent shuddering chills, and always returns at the same hour; thirst only *between* the chill and heat; hot sweat on the face and cold on the rest of the body. *Spigelia.* Chill in the morning at the same hour, chill alternating with either heat or sweat; chill of some parts, with warmth of others; universal morning chill, with concomitant heat; chilliness from the least motion. The chill proceeds from the chest; heat especially on the back; sweat may be offensive (at night), sticky, or cold. *Thuja.* Chill on the *left* side, which is cold to touch; chill after midnight and morning, without thirst, internal chill, with external heat and great thirst; burning heat of the face without redness, dry heat of covered parts, sweat on the uncovered parts of the body, and dry heat on the covered, or the reverse; sweat following the chill immediately, without heat, sweat may be fatty, offensive or sweetish smelling.

There are other remedies which are characterized by the return of their intermittent paroxysms at the same hour, which may be studied where the case in hand is not represented by either of the above with the degree of similarity which will warrant its selection as the required curative. These are *Apis, Cina, Conium, Graphites, Hepar, Mag-mur., Phosphorus, Stan-*

*num*, and *Staphisagria*. It is to be borne in mind where this exact return is met in a case to be treated it is a factor of importance in the control of the choice of the curative.

For heat returning at the same hour, study *Sabadilla, Silicea*, and *Stannum*. The absence of thirst, predominance of the chill, which appears afternoon and evening, while the heat, if any, comes after midnight and morning, is sufficient to characterize *Sabadilla*. With *Silicea* the heat is afternoon, evening, and night. With *Stannum* the chill comes forenoon and evening, the heat afternoon and evening, and perhaps in short, repeated attacks.

Sweat recurring at the same hour is found with *Ant-crud., Bov., Cina, Ignatia, Sabad.*, and *Spig*. For the characteristics of the other elements of the paroxysms caused by these drugs, *Vide ante.*

The time of day of the appearance of the paroxysm is of great importance among the group of elements for which we are to find a simillimum. Often it is decisive, as between two or more similar groups of the right selection. If, for example, one case presents us a group similar to *Ign., Caps*, or *Carbo-v.*, and appears at any other hour of the day than evening, to give either of them will probably only result in disappointment. In referring to this element in our problem of prescribing, we will understand *morning* as beginning at 4 A. M. and ending at 9 A. M.; *forenoon* at 9 A. M. and ending at 12 M.; *afternoon* beginning at 12 M. and ending at 4 P. M.; evening beginning at 4 P. M. and ending at 9 P. M.; night from 9 P. M. to 4 A. M.; *before midnight*, from 9 to 12; *after midnight*, from 12 to 4. With this division in mind we shall find the following medicines characterized by chill in the

*Morning.*—Acon., Agar., Ambr., Anac., Ang., Ant-crud., Ant-tart., Apis, Arn., Ars., Bar., Bell., Bov., Bry., Calad., Calc., Carb-an., Carbo-veg., Caust., Chin., Cina, Cocc., Coff., Coloc., Con., Creos., Cycl., Dros., Euphras., Graph., Hell., Hep., Kali, Led., Lyc., Magn., Magn-mur., Mang., Merc., Mezer., Mur-ac., Nat-c., Natr-mur., Nitr-ac., Nux-mosch., Nux-v., Phos., Phos-ac., Plumb., Puls., Rheum, Rhod., Rhus, Sassap., Sep., Sil., Spig., Staphisag., Sulph., Sulph-ac., Thuja, Verat.

*Forenoon.*—Agar., Alum., Ambr., Am., Ang., Ant-crud., Ant-tart., Arn., Ars., Asar., Bar., Bell., Bov., Bry., Calc., Cann., Carbo-an., Carbo-veg., Cham., Chin., Cycl., Dros., Euphras., Graph., Guai., Kali, Led., Lyc., Mag., Mag-mur., Merc., Mur-ac., Nat., Natr-mur., Nitr., Nit-ac., Op., Par., Petr., Phos., Phos-ac., Plat., Plumb., Puls., Ran-bulb., Rhod., Rhus, Sabad, Sassap., Sep., Sil., Stann., Staph., Stann., Stront., Sulph., Sulph-ac., Thuja, Viol-tr., Zinc.

*Noon.*—Alum., Ant-crud., Arg., Asar., Bov., Bry., Calc., Kali, Lact., Magn., Nat-mur., Nux-v., Phos., Ran-bulb., Stram., Sulph.

*Afternoon.*—Alum., Amm., Amm-mur., Anac., Ang., Ant-crud., Apis, Arg., Arn., Ars., Asaf., Asar., Bar., Bell., Bov., Bry., Calc., Camph., Canth., Carbo-an., Carbo-veg., Caust., Cham., China, Cina, Cocc., Coff., Con., Croc., Dig., Dros., Euphras., Graph., Guai., Hyos., Ignat., Ipecac., Kali, Lach., Lauro., Lyc., Mag., Mag-mur., Mer., Merc., Mez., Natr., Nat-mur., Nitr., Nit-ac., Nux-v., Petrol., Phos., Phos-ac., Puls., Ran-bulb., Rhus, Sabad., Sep., Sil., Spig., Spong., Stann., Staph., Stram., Sulph., Sulph-ac., Thuja, Verat., Zinc.

*Evening.*—Acon., Agar., Alum., Ambra., Amm., Amm-mur., Ant-crud., Ant-tart., Apis, Arg., Arn., Ars., Asar., Aur., Bar., Bell., Bor., Bov., Bry., Calad., Calc., Camph., Canth., Caps., Carbo-an., Carbo-veg., Caust., Cham., Chel., China, Cina, Cocc., Cal., Con., Croc., Cycl., Dulc., Fer., Graph., Guai., Hell., Hepar, Hyos., Ignat., Ipec., Lach., Laur., Led., Lyc., Magn., Mag-mur., Meny., Merc., Merc-corr., Mezer., Mur-ac., Natr., Natr-mur., Nitr., Nit-ac., Nux-mos., Nux-v., Op., Par., Petrol., Phos., Phos-ac., Plat., Plumb., Puls., Ran-bulb., Ran-scel., Rhod., Rhus, Sabad., Sabin., Samb., Sassap., Scill., Sep., Sil., Spig., Spong., Stann., Staph., Stram., Stront., Sulph., Sulph-ac., Thuja, Verat., Vit., Zinc.

*Night.*—Agar., Alum., Ambr., Amm., Amm-mur., Ang., Ant-tart., Arg., Ars., Bar., Bell., Bor., Bov., Bry., Calad., Calc., Canth., Caps., Carbo-an., Carbo-veg., Caust., Cham., China, Can., Creos., Dros., Euphras., Ferr., Hepar, Hyos., Iod., Ipecac., Kali, Laur., Lyc., Mag., Mag-mur., Mang., Merc.,

